# SpaceMed: Immersive interdisciplinary academic training for the future of human space exploration

**DOI:** 10.1113/EP094012

**Published:** 2026-06-27

**Authors:** Damian M. Bailey, Hervé Normand, Gaëlle Quarck, Gilles Clement, Nicolas Bessot, Antoine Langeard, Olivier Etard, Thomas Freret, Urša Ciuha, Anika Friedl‐Werner, Katharina Brauns, Igor Mekjavic, Alexander C. Stahn, Pierre Denise

**Affiliations:** ^1^ Neurovascular Research Laboratory, Faculty of Life Sciences and Education University of South Wales Glamorgan UK; ^2^ Bexorg, Inc. New Haven Connecticut USA; ^3^ COMETE Laboratory, Department of Clinical Physiology University Hospital of Caen Normandy Caen France; ^4^ Department of Automatics, Biocybernetics and Robotics Jožef Stefan Institute Ljubljana Slovenia; ^5^ Berlin Institute of Health, Institute of Physiology, Center for Space Medicine and Extreme Environments Charité‐Universitätsmedizin Berlin, Corporate Member of Freie Universität Berlin, Humboldt‐Universität zu Berlin Berlin Germany

The main hope of a nation lies in the proper education of its youth

Desiderius Erasmus Roterodamus (1466–1536)

## 1 WHY SPACE BIOMEDICINE MATTERS

**FIGURE 1:**
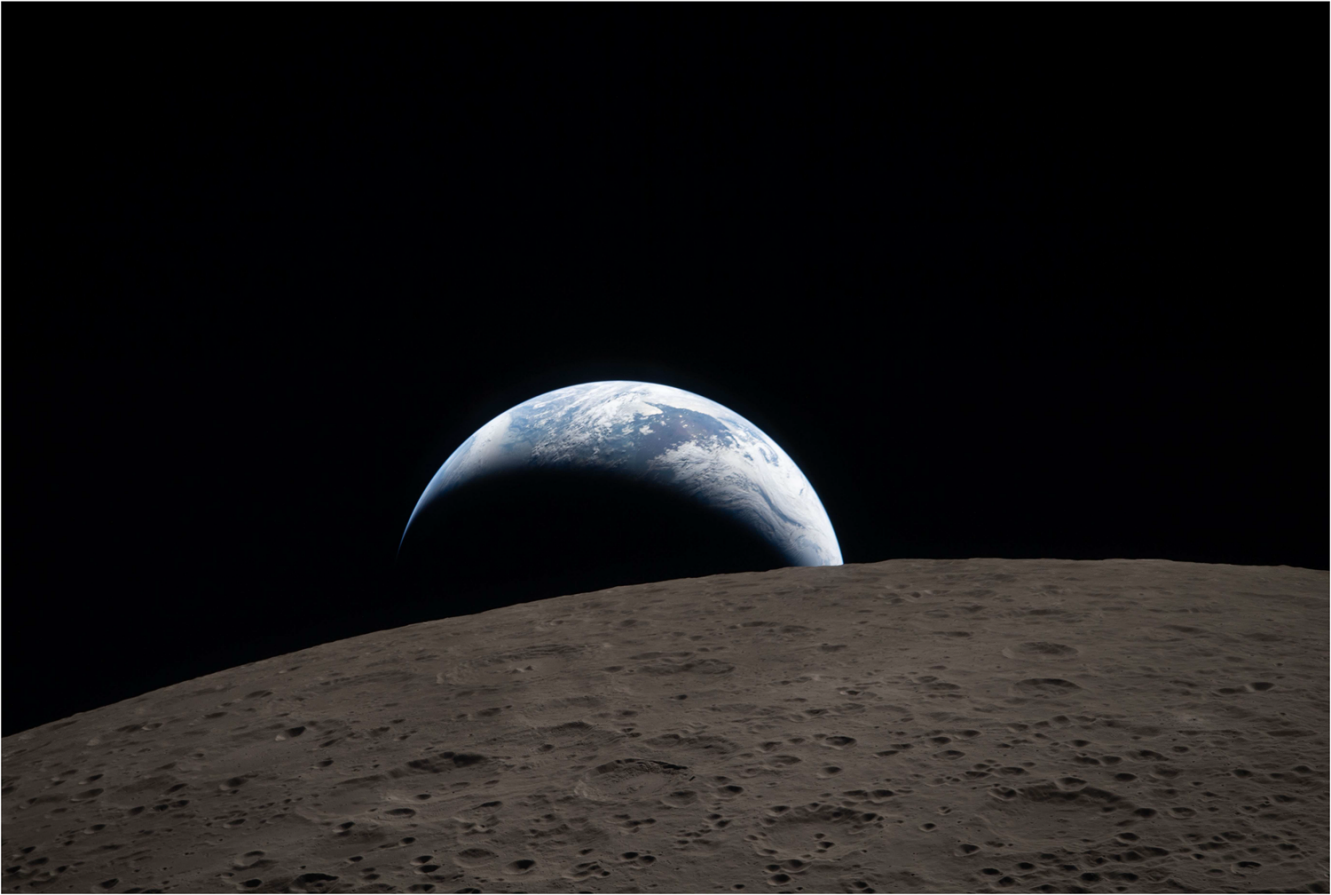
Earthset photographed through the Orion spacecraft window during the Artemis II lunar flyby. The image was captured during the lunar flyby phase of the mission, which went on to carry the crew to a record distance of 252,756 miles (406,771 km) from Earth. Unlike Apollo 13, whose lunar flyby followed an in‐flight technical failure and emergency return trajectory, Artemis II achieved this as part of a planned mission profile. Image credit: NASA.

The recent return of crewed deep‐space exploration has reignited the public imagination. Artemis II, with its lunar flyby (Figure 1) and longer‐term horizon goal of sending humans to Mars, reminds us that the next great era of exploration will be defined not only by rockets and robotics, but by human physiology. Yet as exploration ambitions extend deeper into space, so too does the need to confront a simple truth: there is no environment more physiologically demanding for humans than outer space (Bailey & van Ombergen, 2025c).

Beyond low Earth orbit, astronauts are exposed to the full spectrum of the space exposome: the cumulative sum of environmental, operational and psychosocial stressors encountered across the course of a mission. This includes not only cosmic radiation, isolation and confinement, distance from Earth, hostile closed habitats and altered gravity fields, but also disrupted circadian rhythms, restricted medical capability, operational workload and prolonged separation from terrestrial support systems (Bailey et al., 2025b). Importantly, these stressors rarely act in isolation. Rather, they trigger integrated physiological responses across molecular, cellular, organ‐system and behavioural levels, and it is this multilevel biological embedding of the space environment that may be considered the space integrome (Bailey, 2025a; Bailey et al., 2025b). In this sense, the exposome describes the totality of external mission stressors, whereas the integrome reflects the integrated whole of the physiological responses they evoke, including unanticipated interactions and combinatorial effects that may aggregate to alter risk profiles. What distinguishes the integrome from the exposome concept is that it moves beyond the boundary constraints of focusing on how a single organ system, pathway or gene responds to the space environment, towards a deeper phenotyping of multivariate, integrated responses across the human organism. This more holistic perspective has the potential not only to improve risk stratification, but also to accelerate biomarker discovery, reveal previously unrecognised mechanisms, generate new hypotheses, and ultimately support the development of more targeted and effective countermeasures. Risk is therefore shaped not by any single hazard in isolation, but by the dynamic interplay between mission stressors and the integrated biological responses they provoke, spanning the continuum from acute in‐mission impairment to maladaptation and disease emerging later in life (Bailey, 2025a; Bailey et al., 2025b).

Understanding these risks and developing effective countermeasures is one of the great biomedical challenges of the 21st century. Yet the importance of this work extends far beyond astronauts. Space biomedicine offers a powerful lens through which to understand human resilience, adaptation and breakdown, with direct relevance to ageing, deconditioning, neurovestibular dysfunction, bone and muscle loss, immunodepression, psychological health and healthcare delivery in remote or resource‐limited settings here on Earth (Bailey et al., 2025b; Goswami et al., 2026; Stahn & Kuhn, 2022).

Meeting this challenge requires a new generation of scientists, engineers, clinicians and innovators able to think beyond disciplinary boundaries. Training that generation is therefore an important part of building the field. One example is the Erasmus Mundus Joint master's degree on Physiology and Medicine of Humans in Space and Extreme Environments (SpaceMed: https://www.space‐med.eu/). Its design, structure and the calibre of its students make it a timely and valuable initiative.

## 2 SPACEMED: A MODEL FOR ACADEMIC TRAINING

SpaceMed is a 2‐year, full‐time European Master's programme providing integrated, multidisciplinary training in the physiology and medicine of humans exposed to spaceflight and other extreme environments, each of which presents distinct integrative physiological challenges (Vrdoljak et al., 2026). A particular strength of the programme is its integration of theory and practice. Students learn through classroom teaching, hands‐on laboratory activities and field‐based training. The curriculum spans a multitude of extreme environments including high‐altitude, underwater, cave isolation and thermally challenging environments including heatwave simulation, as well as key spaceflight analogues including parabolic flight, Antarctic habitats and experimental bed‐rest studies. In doing so, it offers students something more valuable than passive knowledge: it gives them physiological intuition, technical confidence and a direct sense of how humans respond when pushed beyond the limits of everyday Earth‐bound life.

The programme is delivered through a partnership between Charité–Universitätsmedizin (Berlin, Germany), Jožef Stefan International Postgraduate School (Ljubljana, Slovenia) and Université de Caen Normandie (Caen, France). SpaceMed was conceived and developed through close collaboration among the three institutions, with the Université de Caen Normandie providing key leadership during its inception. That mutual and interdependent international structure is central to its ethos. Space research is inherently collaborative, multinational and translational, and SpaceMed mirrors that reality by exposing students to different scientific cultures, complementary expertise and a wider ecosystem that includes research institutes, industry and space agencies. For their master's thesis, students can work not only within the partner universities, but also with associated organisations, helping position them at the interface of discovery science, applied physiology and innovation.

## 3 FROM CURRICULUM TO COMPETENCE

**FIGURE 2:**
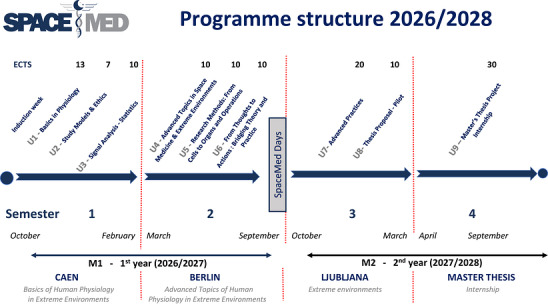
SpaceMed programme structure and content (SpaceMed: https://www.space‐med.eu/).

The scope and organisation of SpaceMed merit attention. Organised across four semesters (Figure 2), students move from foundational principles to advanced application, and from taught content to independent research. This progression is supported by core teaching resources, including (as but one example) the textbook, *Fundamentals of Space Medicine* (Clément, 2025), which provides an important foundation for the lectures in space physiology and medicine.

**FIGURE 3:**
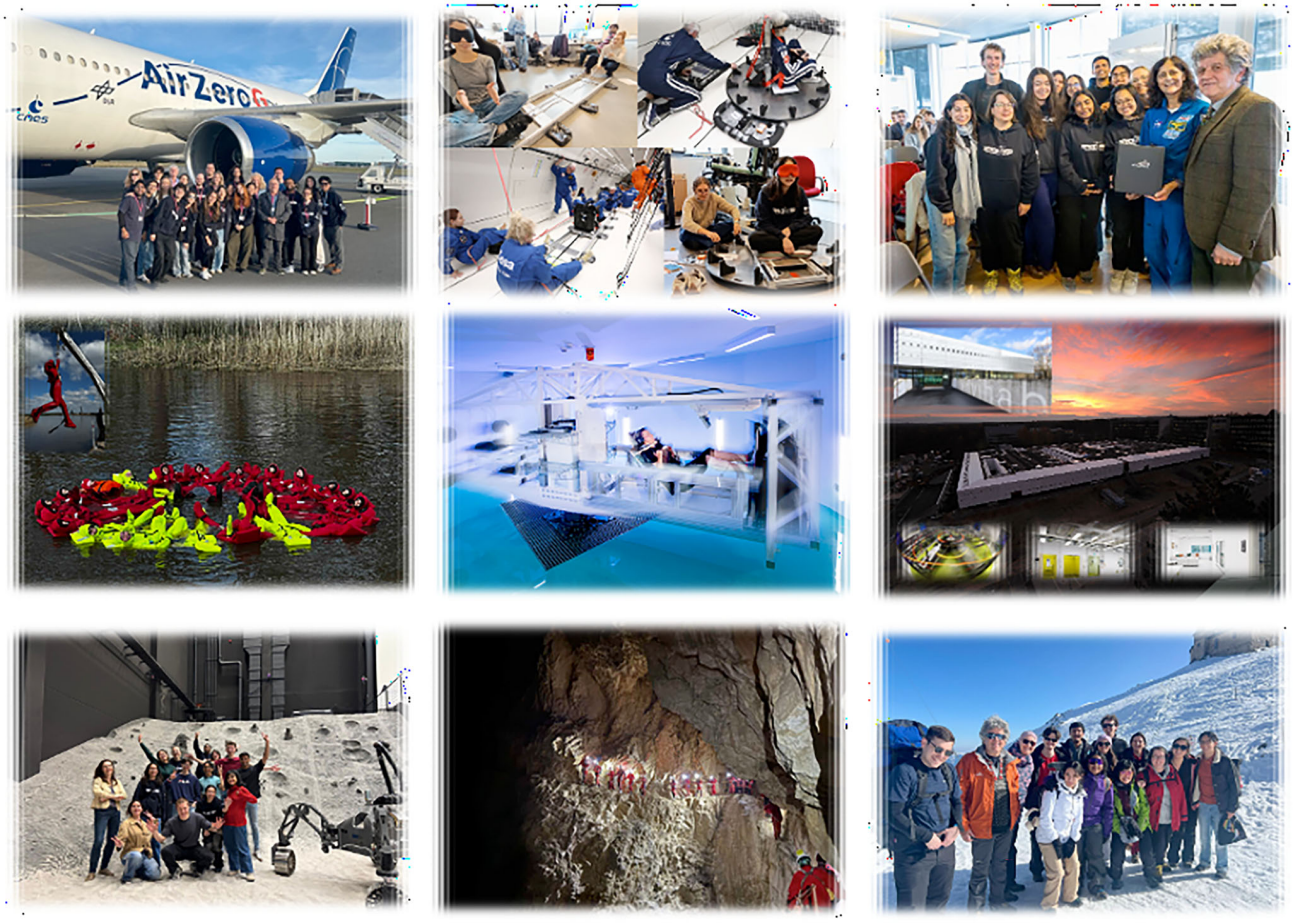
Diversity of practical workshops in extreme physiology. The programme is supported by four SpaceMed ambassadors: ESA astronaut François Clervoy, Canadian astronaut Robert Thirsk, (former) NASA astronaut Sunita Williams and the French Ambassador to Slovenia, Her Excellency Fabienne Runyo. Immersive student training across exploration‐relevant environments and experimental approaches, including parabolic flight in Mérignac, France, with Sunita Williams; cold‐water immersion and rescue training at the Maritimes Kompetenzzentrum (MARIKOM), Elsfleth; the European Space Agency short‐arm human centrifuge at the Olympic Sport Centre Planica near Kranjska Gora, Slovenia; the:envihab facility at the DLR Institute of Aerospace Medicine, Cologne, an advanced medical research platform for studying extreme environmental exposure and countermeasures, including short‐arm centrifugation, environmental chambers, and living and simulation facilities (https://www.dlr.de/en/me/research‐and‐transfer/research‐infrastructure/envihab‐cologne); advanced robotics for medical technology at the German Research Centre for Artificial Intelligence (DFKI); and caving and high‐altitude expedition training. Image credits: A. C. Stahn, K. Bidovec, M. Verč and A. Hodalič. All students featured provided signed consent for the inclusion of their images.

The first semester in Caen, France, establishes the core physiology of extreme environments, covering hypoxia, thermoregulation, gravity, isolation, confinement, hypoactivity and the effects of spaceflight, while also introducing study models, ethics, data acquisition and statistics (Figure 3). The second semester in Berlin, Germany, expands the curriculum into advanced bioastronautics, terrestrial extremes, research methods, data literacy and practical laboratory implementation, while also emphasising science communication and public engagement (Figure 3). The third semester in Ljubljana, Slovenia, focuses on exploration‐relevant physiology, including habitats in extreme environments, sarcopenia, cardiovascular deconditioning, circadian rhythms, sleep, nutrition and space ergonomics, human‐robot interaction/collaboration, alongside pilot work and thesis preparation (Figure 3).

These locations and their respective partners are not incidental, but reflect the specific experimental infrastructure, technical capabilities and specialist expertise available at each host institution and country. Together, their complementary expertise and resources allow students to learn directly from established leaders in the field while engaging with the tools, models and environments that underpin contemporary research in space physiology and medicine. The final semester is devoted to a 6‐month master's thesis or internship, shifting the student from learner to contributor.

The organisation of the programme reflects a deeper philosophy. SpaceMed is not simply about exposing students to exciting and adventurous topics; it is about cultivating genuine competence. Graduates are trained to understand how integrated physiological systems respond to spaceflight and extreme environments, to evaluate ground‐based analogues and countermeasures, to handle complex equipment and advanced analysis, and to communicate their science clearly in both written and oral form. Just as importantly, they develop the broader professional skills the field now demands to tackle the complexities posed by the space exposome: teamwork, initiative, accountability and the ability to work effectively across disciplines. SpaceMed therefore offers more than subject‐specific teaching; it provides students with a practical and intellectually integrated training experience.

An important aim of the Erasmus Mundus programme is also to introduce students to the history and culture of Europe, in keeping with the ideals of Desiderius Erasmus Roterodamus (1466–1536), after whom the programme is named. The multicultural character of the SpaceMed cohorts further fosters mutual respect and a deeper appreciation of the diverse cultures represented within the student body. Importantly, the programme promotes not only student mobility, but faculty mobility as well.

## 4 SPACEMED STUDENT PROFILE AND DIVERSITY

The value of SpaceMed lies not only in its structure, but also in the quality and diversity of its students. This is also reflected in several early programme metrics. For the 2024 intake, SpaceMed attracted 360 applications from 66 countries, from which 12 students were selected; nine received scholarships (€1400/month for 24 months) with full fee waivers, three were self‐funded, and eight were women. For the 2025 intake, 619 applications were received from 71 countries, resulting in the selection of 23 students, of whom 21 received scholarships, two were self‐funded, and 13 were women. For the 2026 intake, 673 applications were received from 79 countries, with 23 students selected (15 women and 8 men), including 22 scholarship recipients and one self‐funded student. Although the first cohort will not formally graduate until the end of September 2026, eight students have already received offers for doctoral positions following completion of the programme.

**FIGURE 4:**
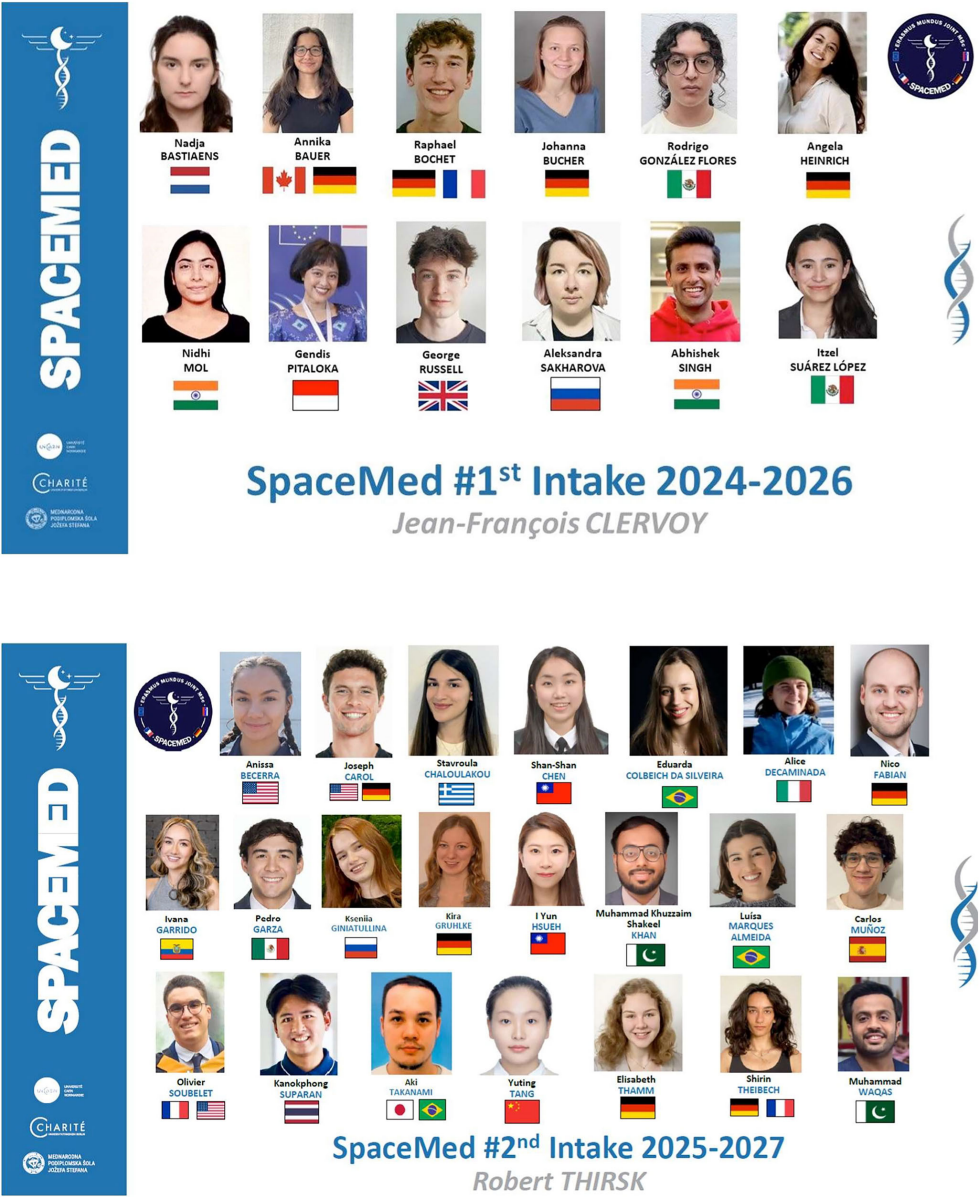
SpaceMed students. All students featured provided signed consent for the inclusion of their names and images.

Following a rigorous selection process, the inaugural cohort, welcomed on 30 September 2024, comprised students from no less than nine countries: Canada, the UK, France, Germany, India, Indonesia, Mexico, the Netherlands and Russia (Figure 4). On 29 September 2025, the programme welcomed its second cohort representing 15 nationalities: Brazil, China, Ecuador, France, Germany, Greece, Italy, Japan, Mexico, Pakistan, Russia, Spain, Taiwan, Thailand and the United States (Figure 4). The third cohort has just been selected from Azerbaijan, Bangladesh, Canada, Ecuador, Egypt, Germany, Indonesia, Italy, Kazakhstan, Mexico, Nigeria, Pakistan, Peru, Ukraine and the United States. This breadth of representation reflects the international and collaborative nature of space exploration, while also bringing a valuable diversity of academic, cultural and professional perspectives to the programme.

What is especially striking is how quickly students begin to think beyond traditional disciplinary silos. Many arrive with backgrounds in nutrition, medicine, physiology, psychology, biochemistry, physiotherapy, engineering, or sports and health and exercise science, but the programme rapidly broadens their horizons. As Stavroula Chaloulakou reflected, her training helped her appreciate not only the practical challenges of astronaut nutrition, but the need for a far more integrated approach and the opportunities this opens across allied disciplines. Likewise, Kanokphong Suparan described the programme as inspiring from a clinical perspective, highlighting the importance of understanding the need for a deeper phenotyping of the space integrome to develop more effective and targeted countermeasures to mitigate risk and improve crew safety and performance, while also benefiting patient health on Earth. These reflections capture something central to SpaceMed: it does not train students to remain shackled to disciplinary lanes, but to connect ideas, methods and applications across them.

This has been observed first‐hand, including through students who have undertaken internships in our laboratories. They have consistently impressed by their maturity, enthusiasm and willingness to engage with complexity. They embrace challenge and exhibit exactly the kind of integrative thinking that the field is likely to require (Bailey et al., 2025b; Stahn et al., 2023). In this respect, the students provide a strong indication of the programme's potential. They are not simply passing through a master's degree. They are emerging members of a community, enriched by the multicultural spirit of Europe, that may help inform the next phase of space physiology and medicine and contribute to addressing the challenges that lie ahead.

SpaceMed is therefore more than a teaching initiative. It provides a strong example of how interdisciplinary training can help prepare the next generation for the challenges of human space exploration. At a time when human space exploration is again gathering momentum, programmes such as this take on particular importance, not least because future progress will depend as much on human capability as on technological innovation. They should also serve as a reminder that Europe must remain committed to supporting such initiatives, and building bridges between different cultures and societies, since Erasmus Mundus programmes are necessarily contract‐based and depend on sustained investment if they are to deliver long‐term strategic value.

## AUTHOR CONTRIBUTIONS

Damian M. Bailey conceived the idea and wrote the first draft of the manuscript with initial input from Hervé Normand and Pierre Denise. All authors edited and approved the final version submitted for publication and agree to be accountable for all aspects of the work in ensuring that questions related to the accuracy or integrity of any part of the work are appropriately investigated and resolved. All persons designated as authors qualify for authorship, and all those who qualify for authorship are listed.

## CONFLICT OF INTEREST

Damian M. Bailey is Editor‐in‐Chief of *Experimental Physiology* and outgoing Chair of the Life Sciences Working Group and outgoing member of the Human Spaceflight and Exploration Science Advisory Committee to ESA. He is a current member of the ESA‐HRE‐Biology Panel and Space Exploration Advisory Committees to the UK and Swedish National Space Agencies.

## GENERATIVE AI STATEMENT

The authors confirm that no artificial intelligence tools, including large language models (LLMs), were used in the drafting or revision of this manuscript. All content was conceived, written and approved solely by the authors.
